# Distribution of Lymph Node Metastases in Esophageal Carcinoma Patients Undergoing Upfront Surgery: A Systematic Review

**DOI:** 10.3390/cancers12061592

**Published:** 2020-06-16

**Authors:** Eliza R. C. Hagens, Mark I. van Berge Henegouwen, Suzanne S. Gisbertz

**Affiliations:** Department of Surgery, Amsterdam University Medical Centers, University of Amsterdam, Cancer Center Amsterdam, 1105AZ Amsterdam, The Netherlands; e.r.hagens@amsterdamumc.nl (E.R.C.H.); s.s.gisbertz@amsterdamumc.nl (S.S.G.)

**Keywords:** esophageal cancer, esophagectomy, lymphadenectomy, lymph node metastases, systematic review

## Abstract

Metastatic lymphatic mapping in esophageal cancer is important to determine the optimal extent of the radiation field in case of neoadjuvant chemoradiotherapy and lymphadenectomy when esophagectomy is indicated. The objective of this review is to identify the distribution pattern of metastatic lymphatic spread in relation to histology, tumor location, and T-stage in patients with esophageal cancer. Embase and Medline databases were searched by two independent researchers. Studies were included if published before July 2019 and if a transthoracic esophagectomy with a complete 2- or 3-field lymphadenectomy was performed without neoadjuvant therapy. The prevalence of lymph node metastases was described per histologic subtype and primary tumor location. Fourteen studies were included in this review with a total of 8952 patients. We found that both squamous cell carcinoma and adenocarcinoma metastasize to cervical, thoracic, and abdominal lymph node stations, regardless of the primary tumor location. In patients with an upper, middle, and lower thoracic squamous cell carcinoma, the lymph nodes along the right recurrent nerve are often affected (34%, 24% and 10%, respectively). Few studies describe the metastatic pattern of adenocarcinoma. The current literature is heterogeneous in the classification and reporting of lymph node metastases. This complicates evidence-based strategies in neoadjuvant and surgical treatment.

## 1. Introduction

Esophageal cancer patients often present with an advanced disease stage, encompassing metastatic lymph nodes or distant metastases. The presence and number of lymph node metastases are among the most important prognostic factors in esophageal carcinoma and are independent predictors for long-term survival [1,2,3,4,5,6].

The location of metastatic lymph nodes depends on tumor histology, primary tumor location, T-stage and neo-adjuvant therapy [7]. The vessels in the dense lymphatic network surrounding the esophagus are complexly aligned and they contribute to a multidirectional spread of lymph node metastases in the abdomen, the mediastinum, and the neck [8,9]. Additionally, ‘skip metastasis’, skipping the first and directly metastasizing into the second or third lymph node echelons, are frequently seen in both esophageal adenocarcinoma and squamous cell carcinoma [10,11]. This contributes to the presence of lymph node metastases at unexpected distant sites, which makes standardization of the extent of the radiation field and lymphadenectomy in the treatment of esophageal cancer difficult. Not surprisingly, the optimal extent of the lymphadenectomy in esophagectomy has been subject of a global debate over the past decades [12,13]. In addition, several classification systems exist, contributing to the heterogeneity in the reporting of studies and clinical practice [14,15].

An extensive lymphadenectomy may result in more post-operative complications, while an insufficient lymphadenectomy carries the risk of understaging and undertreating patients, which may reduce long-term survival [16]. In Western countries, a two-field lymphadenectomy is preferred for distal esophageal adenocarcinomas. Especially in the upper mediastinum, the extent varies considerably among surgeons, centers, and countries [17]. In Asia, where predominantly squamous cell carcinoma is seen, an extensive three-field lymphadenectomy is common practice [12].

Current studies on metastatic lymphatic mapping in esophageal cancer suggest possible dissemination patterns but do not come forward with sufficient evidence to determine the optimal extent of the radiation field and lymphadenectomy. The lack of homogeneity concerning the classification of lymph node stations makes the interpretation of studies difficult and data hard to compare. In other types of cancer, such as pancreatic, breast, and in colon cancer, the standardization of lymphadenectomy has already been established and it has improved oncologic outcome in the long term [18,19,20].

The objective of this review is to identify the locoregional distribution of lymph node metastases in esophageal cancer patients in potentially curable patients, stratified for histology, tumor location, and T-stage. An outline on metastatic lymphatic distribution patterns may contribute to a uniform worldwide staging system and to the standardization of the extent of the radiation field and lymphadenectomy in esophageal carcinoma. 

## 2. Methods

A review protocol was developed based on the Preferred Reporting Items for Systematic Reviews and Meta-Analysis (PRISMA) statement (www.prisma-statement.org) and was registered in the International Prospective Register of Systematic Reviews (PROSPERO) database (CRD42018102804). PubMed and Embase were searched on 22 July 2019. The following terms were used (including synonyms and closely related words) as index terms or free-text words: ‘esophageal cancer (including junctional carcinomas)’ and ‘lymph node metastasis’ and ‘lymphadenectomy’. The full search strategies for PubMed and Embase.com can be found in Appendix A. Duplicate articles were excluded.

Articles were screened by two independent researchers (EH, SSG) in two stages: screening of titles and abstracts followed by the retrieval and screening of full-text articles. Inclusion criteria were as follows: studies describing a complete 2- or 3-field lymphadenectomy by transthoracic esophagectomy, the prevalence of patients with lymph node metastases per lymph node station is given or can be calculated, data is separately reported for adenocarcinoma and/or squamous cell carcinoma and tumor location. We excluded studies describing surgery following neo-adjuvant therapy (because the distribution may be different after neo-adjuvant chemo(radio)therapy), imaging studies, case reports, conference abstracts and reviews, and papers in another language than English or Dutch.

### 2.1. Data Extraction

The primary endpoint is the metastatic rate of lymph node metastases in esophageal carcinoma per lymph node station or region. When available, the following variables were extracted from the included studies: year of publication; country; study design; inclusion period; lymph node classification system used (JES, Japan Esophageal Society; AJCC, American Joint Committee on Cancer; other; none); description of how detailed lymph node regions or stations are described and reported; number of patients; patient characteristics (gender, age); tumor histology; tumor location (upper thoracic esophagus, middle thoracic esophagus, lower thoracic esophagus or gastroesophageal junction (GEJ)); c/pT-stage; c/pN-stage; type of surgical approach; lymphadenectomy (complete 2- or 3- field); use of immunohistochemistry staining; prevalence of lymph node metastases per lymph node station; number of patients with lymph node metastases; overall percentage of positive lymph nodes; number of patients per lymph node location with resected nodes and positive nodes in that station or region. Methodological quality was assessed using the Methodological Index for Non-Randomized Studies (MINORS) checklist [21].

### 2.2. Statistical Analysis

Descriptive statistics summarized the characteristics of included studies, patient characteristics, and the outcomes of each included study. Since not all studies used the same classification or definition for lymph node stations, and some studies only reported lymph node data for different regions instead of stations, we combined the two mostly used systems (JES and AJCC) and grouped lymph node stations into five regions: cervical, upper mediastinal, middle mediastinal, lower mediastinal, and abdominal (Table 1). Studies with a reported number of patients with metastatic lymph nodes per station were pooled separately from the studies only describing data per region. The prevalence of patients with lymph node metastases per station or region were calculated by summarizing all the patients with lymph node metastases per lymph node station or region and dividing them by the sum of all patients who had a lymph node dissection in this station or region. Results were stratified for tumor histology and primary tumor location. Finally, studies describing the lymphatic distribution pattern according to the pT-stage were pooled and described separately.

## 3. Results

**Figure 1:**
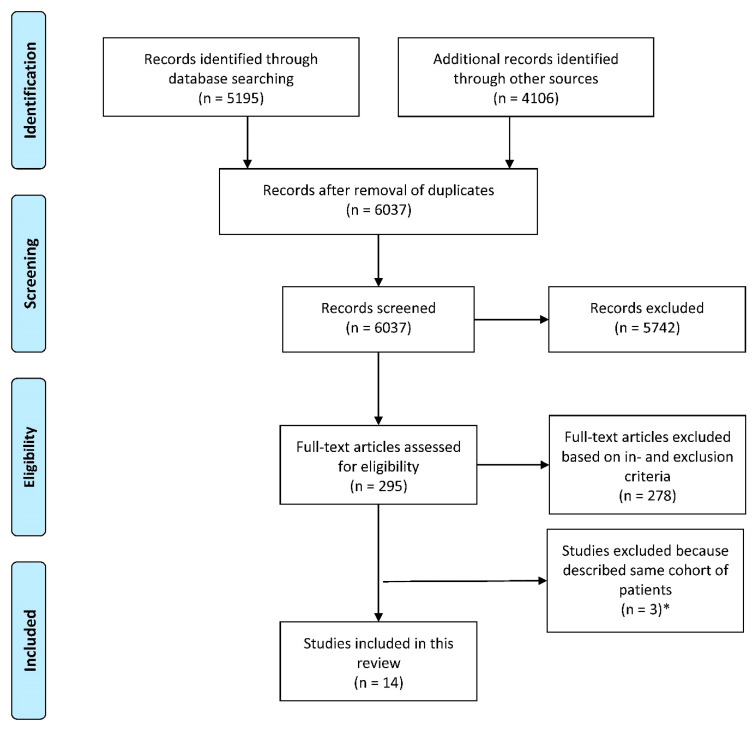
Flow-chart of study selection. * Three studies by Chen and colleagues and two by Li and colleagues described (partly) the same cohort of patients. Therefore, only one study of Chen et al. and one study of Li et al. were included. For both, this was the study where most of the lymph node stations and/or regions were described.

Details of the literature search and study selection are shown in Figure 1. Fourteen studies met the inclusion criteria and were included in this review [7,22,23,24,25,26,27,28,29,30,31,32,33,34]. Characteristics of the included studies are presented in Table 2. A total of 8952 patients were evaluated, including 409 (5%) with an adenocarcinoma and 8543 (95%) with a squamous cell carcinoma. Among all patients with a squamous cell carcinoma, 726 (9%) patients had a tumor located in the upper thoracic esophagus, 5130 (60%) patients had a tumor in the middle thoracic esophagus and 2687 (31%) had a tumor in the lower thoracic esophagus. None of these studies described patients with a cervical or GEJ squamous cell carcinoma. For adenocarcinoma, 32 (8%) tumors were located in the distal esophagus and 377 (92%) were located at the GEJ. The c/pT stage varied among studies, but most of the patients had a c/pT3 tumor. Details of the study populations can be found in Table 2 and Table 3.

### 3.1. Reporting Standard

Standard reported lymphadenectomy differed among studies. In 1 study, patients underwent a 2-field lymphadenectomy, in 9 studies, patients underwent a 3-field lymphadenectomy, and in 4 studies, both procedures were included, resulting in a different nodal yield per study. In addition, the definition of anatomical locations of lymph node stations differed amongst studies; 1 study used AJCC, 5 used JES, and 8 did not use a standard classification system. Moreover, 6 studies described the prevalence of lymph node metastases per lymph node station, 5 only described the prevalence of lymph node metastases per region, and 3 reported a combination of both. The reported regions and stations also varied among studies (Table 2). Some studies described for some stations both sides together, and other studies separated left and right. One study combined tumor locations when reporting the number of patients per lymph node station and could therefore not be pooled with the others studies [7].

### 3.2. Distribution Pattern for Esophageal Squamous Cell Carcinoma

**Figure 2:**
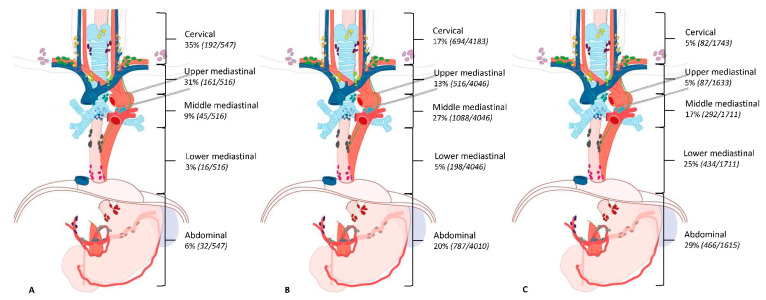
Prevalence of lymph node metastases per tumor location in patients with squamous cell carcinoma. (**A**) = Upper thoracic esophageal tumor, (**B**) = Middle thoracic esophageal tumor, (**C**) = Distal thoracic esophageal tumor. Data presented as percentage of patients with lymph node metastases in this region.

Eleven studies [7,22,24,26,27,28,29,30,31,32,34] described the location of lymph node metastases in patients with a squamous cell carcinoma (*n* = 8543). Table 4 shows the prevalence of lymph node metastases per lymph node station among the seven studies [22,26,28,29,30,31,34] that reported data per lymph node station per tumor location. For patients with an upper thoracic tumor, lymph node metastases are most frequently seen along the right recurrent nerve (60%) and cervical paraesophageal lymph nodes (right 34% and left 22%). For patients with a middle thoracic tumor, the prevalence of lymph node metastases was highest along the right recurrent nerve (23%), right cervical paraesophageal lymph nodes (24%), and middle thoracic paraesophageal lymph nodes (23%). The lymph nodes along the left gastric artery (28%) and lower thoracic esophagus (23%) had the highest prevalence of lymph node metastases in patients with a tumor in the lower thoracic esophagus. Six studies [24,26,27,28,30,32] described the location of the lymph node metastases per region. The results of these studies are shown in Figure 2.

### 3.3. Distribution Pattern for Esophageal Adenocarcinoma

Four studies [7,23,25,33] described the location of lymph node metastases in patients with an adenocarcinoma (*n* = 409). One study [33] described the prevalence of metastatic lymph nodes per lymph node station; this was for GEJ tumors (Table 4). Lymph node stations with the highest prevalence of patients with metastatic lymph nodes were lymph nodes along the left gastric artery (48%), lesser curvature (29%), splenic artery (26%), right paracardial lymph nodes (26%), and subcarinal lymph nodes (25%).

Two studies [23,25] described the location of lymph node metastases in regions. Pooled numbers show that for patients with a GEJ tumor, 20% (4 out of 10) had lymph node metastases in the cervical region and 25% (31 out of 124) had metastases in the abdominal lymph node stations (other regions were not reported). For patients with an adenocarcinoma of the lower thoracic esophagus, 35% (6 out of 17) had lymph node metastases in the cervical region, 71% (12 out of 17) had lymph node metastases in the lower mediastinal region, and 71% (12 out of 17) had lymph node metastases in the abdominal region.

One study [7] combined patients with a tumor of the distal esophagus and GEJ. In this study, a prevalence of 30% in the periesophageal lymph nodes, 37% in the paracardial lymph nodes, 35% in the perigastric lymph nodes, and 14% in the celiac axis was reported.

### 3.4. Distribution of LN Metastases in Relation to pT-Stage

Three studies [24,27,30] stratified the prevalence of nodal metastases per pT-stage. All three studies described patients with squamous cell carcinoma. Table 5 shows the rate of patients with lymph node metastases per region, divided into four groups: patients with pT1-2 and pT3-4 and patients with pT1 and pT2-4. Among patients with higher T-stages, a higher prevalence of lymph node metastases is seen per region, while the distribution remained similar.

## 4. Discussion

This study describes the sites of lymph node metastases in esophageal cancer patients according to the histology and primary tumor location based on literature published before July 2019. This is the first study systematically combining available evidence on lymph node metastases pattern, contributing toward revealing the lymphatic metastatic pattern of esophageal carcinoma. This study showed that both squamous cell carcinoma and adenocarcinoma metastasize to cervical, thoracic, and abdominal lymph node stations, regardless of the location of the primary tumor.

### 4.1. Lymphatic Distribution Pattern for Squamous Cell Carcinoma

Multiple studies have attempted to define the lymph node metastases pattern in esophageal cancer [7,22,23,25,26,27,30,31,32,33]. Most of these studies describe squamous cell carcinoma only. The available evidence of lymph node metastases pattern in adenocarcinoma of the esophagus is scarce, and the available literature for both tumor types is very heterogeneous. Whereas some studies report data per lymph node station, others report per region. To make this even more complex, not all studies adhere to the same boundaries of lymph node regions and not all studies use the same anatomical definition of lymph node stations. To define anatomical sites on lymph node stations, some use standardized classification systems such as the AJCC or JES, while others do not use any standardized classification system. Moreover, not all studies report the exact extent of lymphadenectomy. In addition, some studies excluded from this review combined patients with adenocarcinoma and squamous cell carcinoma and/or different tumor locations, which makes data hard to interpret, since these factors could influence the distribution pattern [35,36,37] All together, these factors make comparing available evidence on the lymph node metastases distribution in esophageal cancer difficult.

For upper, middle, as well as lower thoracic esophageal squamous cell carcinoma, the stations around the esophagus are among those with the highest prevalence of lymph node metastases. However, not only lymph node stations in the same region as the tumor have a high prevalence of nodal metastases; stations in different regions are affected as well. For example, 13% of the patients with a lower esophageal tumor have right cervical lymph node metastases.

### 4.2. Lymphatic Distribution Pattern for Adenocarcinoma

For adenocarcinoma, although data are more limited, similar results are seen. One-quarter (25%) of patients with a GEJ adenocarcinoma had middle thoracic paraesopahgeal lymph node metastases, and when looking at zones, 20% of the patients with a GEJ adenocarcinoma had lymph node metastases in the cervical zone. An explanation could be the presence of an extensive lymphatic network in the submucosa and even in the lamina propria of the esophagus, with both intramural and longitudinal lymphatic drainage. The longitudinal nature of this network explains the variation in anatomic sites of lymph node metastases [38,39,40]. Another result of the complexity of the lymphatic vessel system is the phenomenon of skip metastases [37]. Skip metastases are distant lymph nodes with metastatic involvement, without tumor infiltration in the regional lymph nodes, and they are more often seen in early tumors [10]. It is unclear what the exact clinical value is since the literature is conflicting on the prognostic relevance [41,42,43].

Three studies [24,27,30] described lymph node metastases per lymph node station in relation to pT-stage in patients with squamous cell carcinoma. An increased prevalence of lymph node metastases is seen per region in patients with a higher pT-stage, while the distribution remained similar. These are small numbers; nevertheless, the literature points out that a higher T-stage is associated with more lymph node metastases [44].

It should be pointed out that this study defines the lymph node metastases pattern based on patients without neoadjuvant treatment because lymph node involvement may differ after neoadjuvant chemo(radio)therapy [45]. Currently, neoadjuvant chemoradiation or perioperative or neoadjuvant chemotherapy is the standard of care in most countries. This makes our results less applicable to current surgical patients, and one of the main questions for the future is whether the lymphadenectomy strategy should be based on the pattern of lymph node metastases before neoadjuvant treatment or after neoadjuvant treatment. However, the location of lymph node metastases in untreated esophageal cancer patients tells us more about the behavior of the disease, and this is fundamental, as this allows for accurately defining neoadjuvant treatment strategies by targeting high-risk regions for lymph node metastases in patients with specific characteristics. A recent study showed that after neoadjuvant chemoradiotherapy, almost half of the patients in that cohort had lymph node metastases outside the radiation field, indicating that the current radiation fields are not sufficient [46]. Although, it should be noted that radiotherapy to an elective nodal area (both metastatic and non-metastatic) does not guarantee a better outcome [47].

There are some limitations of the present study. Firstly, as previously mentioned, studies were very heterogeneous in lymph node dissection and the reporting of anatomical sites of nodal metastases. This heterogeneity might have made our pooled results less reliable. Moreover, not all studies could be pooled per station (since they only described lymph node regions) and vice versa. The inclusion of different studies in Table 2 and Table 4 makes displayed percentages slightly different. In addition, few of the studies subdivided patients for T-stage and location of the primary tumor, whilst it has been proven that these factors influence lymph node metastases [26,48,49].

If we want to determine the exact distribution pattern of esophageal cancer, large well-designed prospective studies are needed. One initiative of such a study is the multinational prospective TIGER study (ClinicalTrials.gov Identifier: NCT03222895) [50].

## 5. Conclusions

Both esophageal squamous cell carcinoma and adenocarcinoma are aggressive diseases that can metastasize to cervical, thoracic, as well as abdominal lymph node stations, regardless of the location of the primary tumor. The prevalence of patients with metastatic lymph nodes per station and region could be determined for squamous cell carcinoma. However, few studies described the distribution of lymph node metastases for esophageal adenocarcinoma, and the data for both tumor types was very heterogeneous. This complicates evidence-based treatment strategies in both neoadjuvant (radiation field) and surgical (lymphadenectomy) treatment. Well-designed prospective studies are needed to determine the exact lymphatic distribution pattern of esophageal cancer.

## Figures and Tables

**Table 1:** Combination of JES and AJCC stations and five lymph node regions.

Node Region	Station Number (JES)	Name of Station (JES)	Station Number (AJCC)	Name of Node Station (AJCC)
Cervical	104R	Right supraclavicular lymph nodes		
	104L	Left supraclavicular lymph nodes		
	101R	Right cervical paraesophageal lymph nodes	1R	Right lower cervical paratracheal lymph nodes
	101L	Left cervical paraesophageal lymph nodes	1L	Left lower cervical paratracheal lymph nodes
	102	Deep cervical lymph nodes		
	103	Peripharyngeal lymph nodes		
Upper mediastinal	105	Upper thoracic paraesophageal lymph nodes	8up	Posterior mediastinal lymph nodes
	106preR	Right pretracheal lymph nodes		
	106preL	Left pretracheal lymph nodes		
	106recR	Right recurrent nerve lymph nodes	2R	Right and left upper paratracheal nodes (including lymph nodes along the recurrent laryngeal nerve and the cervical paratracheal lymph nodes)
	106recL	Left recurrent nerve lymph nodes	2L + 4L	Left upper paratracheal nodes + Left lower paratrachal lymph nodes
	106tbR	Right tracheobronchial lymph nodes		Right lower paratrachal lymph nodes
	106tbL	Left tracheobronchial lymph nodes	4L	Left lower paratrachal lymph nodes
Middle mediastinal	107	Subcarinal lymph nodes	7	Subcarinal lymph nodes
	108	Middle thoracic paraesophageal lymph nodes	8M	Middle thoracic paraesophageal lymph nodes
	109R	Right main bronchus lymph nodes	7	Subcarinal lymph nodes
	109L	Left main bronchus lymph nodes	7	Subcarinal lymph nodes
Lower mediastinal	110	Lower thoracic paraesophageal lymph nodes	8Lo	Lower thoracic paraesophageal lymph nodes
	111	Supradiaphragmatic lymph nodes	15	Diaphragmatic lymph nodes
	112	Posterior mediastinal lymph nodes	9	Pulmonary ligament lymph nodes
Abdominal lymph node stations	1	Right paracardial lymph nodes	16	Paracardial lymph nodes
	2	Left paracardial lymph nodes	16	Paracardial lymph nodes
	3	Lesser curvature lymph nodes	17	Lymph nodes along the left gastric artery
	4	Lymph nodes along the greater curvature		
	7	Lymph nodes along the left gastric artery	17	Lymph nodes along the left gastric artery
	9	Celiac lymph nodes	20	Celiac lymph nodes
	8	Lymph nodes along the common hepatic artery	18	Lymph nodes along the common hepatic artery
	11	Splenic artery lymph nodes	19	Splenic artery lymph nodes
	19	Infradiaphragmatic lymph nodes	16	Paracardial lymph nodes

JES = Japan Esophageal Society; AJCC = American Joint Committee on Cancer; Lymph node stations 5, 6, 10, and 12 to 20 of the JES classification not described in this review.

**Table 2:** Characteristics of included studies.

No	First Author	Year of Publication	Study Design	Country	Number of Patients	Inclusion Period	2- or 3-Field Lymphadenectomy	Lymph Node Classification System Used	Use of Immunohistochemistry Staining	MINORS Score	How Detailed Are the Locations of Lymph Node Metastases Described?	How Are the Locations of Nodal Metastases Reported?
1	S. Sharma [22]	1994	Retrospective study	Japan	70	1985–1991	3-field	No standard classification used	NR	10	Description of several stations in cervical, thoracic, and abdominal regions.	Number of patients with resected and positive lymph nodes reported per station.
2	C. van de Ven [23]	1999	Prospective observational study	Belgium	37	1994–1998	3-field	No standard classification used	NR	11	Description of several stations in cervical, thoracic, and abdominal regions.	Number of patients with resected and positive lymph nodes reported per region.
3	H. Igaki [24]	2001	Retrospective study	Japan	96	1986–1998	3-field	No standard classification used	NR	8	No description of stations. Cervical, upper mediastinal, middle mediastinal, lower mediastinal, perigastric, and celiac regions described.	Number of patients with resected and positive lymph nodes reported per region. Numbers per station were not provided.
4	S.M. Dresner [25]	2001	Retrospective study	United Kingdom	104	1996–1999	2-field	No standard classification used	NR	10	Description of stations in lower thoracic and abdominal regions.	Number of patients with resected and positive lymph nodes reported for the abdominal region. Numbers per station were not provided.
5	J. Chen [26]	2009	Retrospective study	China	1850	1993–2006	3-field	JES	H&E	12	Description of stations according to JES in cervical, upper mediational, middle mediastinal, lower mediastinal and abdominal region.	Number of patients with resected and positive lymph nodes reported per region and station.
6	Y. Tachimori [27]	2011	Retrospective study	Japan	356	2001–2005	3-field	No standard classification used	NR	10	No description of stations. Cervical, upper mediastinal, middle mediastinal, lower mediastinal, perigastric and celiac regions described.	Number of patients with resected and positive lymph nodes reported per region. Numbers per station were not provided.
7	C. Castoro [7]	2011	Retrospective study	Italy	248	1992–2007	2-field and 3-field	No standard classification used	H&E and PAS	11	Description of stations in cervical, thoracic and abdominal regions.	Number of patients with resected and positive lymph nodes reported per station.*
8	H. Li [28]	2012	Retrospective study	China	200	2000–2010	3-field	No standard classification used	H&E	11	No description of stations. Cervical, mediastinal, recurrent laryngeal nerve and abdominal regions described.	Number of patients with resected and positive lymph nodes reported per region and the recurrent laryngeal nerve station.
9	S. Kosugi [29]	2013	Retrospective study	Japan	86	1992–2011	3-field	JES	NR	11	Description of stations according to JES in cervical, upper mediational, middle mediastinal, lower mediastinal, perigastric, and suprapancreatic regions.	Number of patients with resected and positive lymph nodes reported per station.
10	J. Cheng [30]	2013	Retrospective study	China	1893	2003–2011	2-field and 3-field	JES	H&E	9	Description of stations according to JES in cervical, upper mediational, middle mediastinal, lower mediastinal, and abdominal region.	Number of patients with resected and positive lymph nodes reported per region and per a selected number of stations.
11	Z. Lin [31]	2016	Prospective observational study	China	260	2009–2013	3-field	AJCC	H&E	13	Description of stations according to AJCC in the thoracic and abdominal region.	Number of patients with resected and positive lymph nodes reported per station.
12	Y. Dong [32]	2015	Retrospective study	China	3587	2000–2014	2-field and 3-field	JES	NR	10	Description of stations according to JES in the cervical, upper mediastinal, middle mediastinal, lower mediastinal, and abdominal regions.	Number of patients with resected and positive lymph nodes reported per region. Numbers per station were not provided.*
13	X. Duan [33]	2017	Retrospective study	China	136	2014–2016	2-field and 3-field	No standard classification used	NR	11	Description of stations in thoracic and abdominal regions.	Number of patients with resected and positive lymph nodes reported per station.
14	S. Park [34]	2018	Prospective observational study	Korea	29	2014–2018	3-field	JES	H&E	10	Description of stations according to JES in the cervical, upper mediastinal, middle mediastinal, lower mediastinal, and abdominal regions.	Number of patients with resected and positive lymph nodes reported per station.

Studies are shown in chronological order; JES = Japan Esophageal Society; AJCC = American Joint Committee on Cancer; * Partly combined locations of the tumor in displayed data; H&E = hematoxylin & eosin; PAS = periodic acid-Schiff; NR = not reported; MINORS score ranges from 0 to 16 for non-comparative studies with 16 being the ideal score.

**Table 3:** Study population characteristics.

No	First Author	Sex, Male *n* (%)	Age, in years	Histology	T-Stage *, *n* (%)	N-Stage *, *n* (%)	Location of the Tumor, *n* (%)	Surgical Approach	Number of Dissected Lymph Nodes per Patient	Percentage of Patients with Lymph Node Metastases	Overall Percentage of Positive Lymph Nodes
1	S. Sharma [22]	62 (89)	mean 58.5	SCC	pT1 11 (16) pT2 12 (17) pT3 46 (65) pT5 1 (2)	pN0 20 (29) pN1 50 (71)	UTE 10 (14) MTE 37 (53) LTE 23 (33)	Open procedures	mean 82	71% (50/70)	4% (208/5720)
2	C. van de Ven [23]	NR	NR	AC	cT3	NR	LTE 17 (46) GEJ 20 (54)	Open procedures	mean 60, SD 17	NR	14% (323/2240)
3	H. Igaki [24]	85 (97)	mean 62 [range 42–86]	SCC	pT1 27 (28) pT2 16 (16) pT3 53 (56)	pN0 36 (38) pN1 60 (62)	LTE	Open procedures	NR	66% (63/96)	NR
4	S.M. Dresner [25]	91 (88)	mean 63 [range 30–78]	AC	NR	NR	GEJ	Open procedures	median 22 [range 11–57]	70% (73/104)	21% (508/2476)
5	J. Chen [26]	1351 (73)	median 55 [range 27–54]	SCC	cT1 109 (6) cT2 348 (19) cT3 1215 (65) cT4 178 (10)	NR	UTE 289 (16) MTE 1381 (74) LTE 180 (10)	Open procedures	mean 26 [range 15–71]	58% (1081/1850)	9% (4350/47470)
6	Y. Tachimori [27]	314 (88)	mean 63 [range 41–80]	SCC	pT1 127 (36) pT2 40 (11) pT3 183 (51) pT4 6 (2)	pN0 110 (31) pN1 116 (33) pN2 81 (23) pN3 49 (13)	UTE 55 (15) MTE 173 (49) LTE 128 (36)	Open procedures	NR	NR	NR
6	C. Castoro [7]	327 (81)	median 63 [IQR 56–70]	SCC 116 (47) AC 132 (53)	cT1 5 (2) cT2 42 (17) cT3 201 (81)	cN0 107 (43) cN1 141 (57)	UTE (all SCC) 25 (10) MTE (all SCC) 50 (20) LTE (AC 15, SCC 41) 56 (23) GEJ (all AC) 117 (47)	Open procedures	AC median 19.5 [IQR 15–27] SCC median 16 [IQR 12–21]	AC 54% (63/116) SCC 67% (88/132)	NR
8	H. Li [28]	163 (82)	mean 57, SD 9	SCC	pT1 18 (9) pT2 45 (23) pT3 114 (56) pT4 23 (12)	NR	UTE 31 (15) MTE 137 (69) LTE 32 (16)	Open procedures	NR	NR	NR
9	S. Kosugi [29]	78 (91)	mean 60, SD 7	SCC	pT1a 7 (8) pT1b 75 (87) pT2 4 (5)	pN0 48 (56) pN1 31 (36) pN2 6 (7) pN3 1 (1)	UTE 17 (20) MTE 59 (69) LTE 10 (11)	Open procedures	NR	47% (40/86)	NR
10	J. Cheng [30]	1474 (78)	< 40: 1% 41–59: 48% ≥60: 51%	SCC	cTis 10 (1) cT1 103 (5) cT2 345 (18) cT4 1173 (62) cT4 262 (14)	NR	UTE 82 (4) MTE 1266 (67) LTE 545 (29)	Open procedures	mean 13	46% (865/1893)	NR
11	Z. Lin [31]	59 (23)	median 61 [IQR 52–67]	SCC	pT1 30 (11) pT2 44 (17) pT3 164 (63) pT4 22 (9)	pN0 119 (46) pN1 67 (25) pN2 54 (21) pN3 20 (8)	UTE 28 (11) MTE 173 (67) LTE 59 (22)	Open procedures	median 35 [IQR 25–46]	54% (141/260)	15% (316/2097)
12	Y. Dong [32]	2536 (72)	median 61	SCC	pT1 435 (14) pT2 935 (25) pT3 1992 (55) pT4 225 (6)	pN0 2223 (62) pN1 1233 (34) pN2 98 (3) pN3 33 (1)	UTE 189 (5) MTE 1837 (51) LTE 1561 (44)	Hybrid procedures	mean 20 [range 16–50]	38% (1.364/3587)	4% (2870/71740)
13	X. Duan [33]	128 (95)	mean 63, SD 9	AC	pT1-2 17 (13) pT3-4 119 (87)	pN0 44 (32) pN1 64 (47) pN2 21 (15) pN3 7 (6)	GEJ	Open procedures	mean 15	68% (92/136)	21% (431/2083)
14	S. Park [34]	26 (90)	mean 63, SD 7	SCC	cT1	cN0 25 (86) cN1 4 (14)	MTE 17 (59) LTE 12 (41)	Robot-assisted procedures	mean 55, SD 17	86% (25/29)	NR

Studies are shown in chronological order; SCC = squamous cell carcinoma; AC = adenocarcinoma; GEJ = gastroesophageal junction; CE = Cervical esophagus; UTE = Upper thoracic esophagus; MTE = middle thoracic esophagus; LTE = lower thoracic esophagus; NR = not reported; SD = standard deviation; IQR = interquartile range; * either pathological or clinical T and N-stage, based on what is reported in the paper.

**Table 4:** Prevalence of lymph node metastases per lymph node station.

Lymph Node Station	Squamous Cell Carcinoma												Adenocarcinoma			
	Upper Thoracic Esophagus				Middle Thoracic Esophagus				Lower Thoracic Esophagus				Gastroesophageal Junction			
**Cervical region**																
Right supraclavicular lymph nodes	9%	34	/	381	10%	266	/	2684	13%	100	/	748	NR			
Left supraclavicular lymph nodes	5%	14	/	299	5%	67	/	1418	3%	6	/	203	NR			
Right cervical paraesophageal lymph nodes	34%	103	/	299	24%	345	/	1418	10%	20	/	203	NR			
Left cervical paraesophageal lymph nodes	22%	65	/	299	11%	152	/	1418	4%	8	/	203	NR			
Right deep cervical lymph nodes	2%	5	/	289	<1%	4	/	1381	0%	0	/	180	NR			
Left deep cervical lymph nodes	2%	5	/	289	1%	8	/	1381	0%	0	/	180	NR			
Peripharyngeal lymph nodes	1%	2	/	289	<1%	1	/	1381	0%	0	/	180	NR			
**Upper mediastinal region**																
Upper thoracic paraesophageal lymph nodes	10%	40	/	388	6%	163	/	2811	3%	7	/	214	NR			
Right pretracheal lymph nodes	12%	43	/	371	6%	81	/	1381	2%	3	/	180	NR			
Left pretracheal lymph nodes	9%	28	/	299	7%	101	/	1418	2%	4	/	203	NR			
Right recurrent nerve lymph nodes	60%	6	/	10	23%	15	/	66	15%	8	/	52	NR			
Left recurrent nerve lymph nodes	11%	32	/	289	7%	102	/	1410	3%	7	/	209	NR			
Tracheobronchial lymph nodes	12%	10	/	82	12%	17	/	145	6%	3	/	49	NR			
**Middle mediastinal region**																
Subcarinal lymph nodes	8%	32	/	398	18%	517	/	2913	14%	121	/	836	25%	1	/	4
Middle thoracic paraesophageal lymph nodes	5%	20	/	388	23%	595	/	2542	21%	170	/	804	2%	1	/	45
Right main bronchus lymph nodes	<1%	1	/	289	2%	24	/	1410	2%	4	/	209	0%	0	/	10
Left main bronchus lymph nodes	1%	2	/	289	3%	37	/	1410	2%	5	/	209	6%	2	/	31
**Lower mediastinal region**																
Lower thoracic paraesophageal lymph nodes	3%	12	/	388	8%	221	/	2851	23%	184	/	809	10%	10	/	96
Supradiaphragmatic lymph nodes	0%	0	/	306	<1%	3	/	1504	5%	39	/	778	0%	0	/	5
Posterior mediastinal lymph nodes	3%	10	/	316	7%	102	/	1545	5%	14	/	259	0%	0	/	17
**Abdominal region**																
Right paracardial lymph nodes	1%	2	/	199	3%	48	/	1447	12%	27	/	232	26%	33	/	128
Left paracardial lymph nodes	3%	10	/	299	7%	201	/	2684	13%	97	/	748	37%	48	/	131
Lesser curvature lymph nodes	3%	9	/	299	10%	273	/	2713	11%	89	/	777	29%	37	/	127
Lymph nodes along the greater curvature	0%	0	/	289	<1%	1	/	1381	0%	0	/	180	12%	5	/	41
Lymph nodes along the left gastric artery	4%	11	/	299	16%	238	/	1528	28%	76	/	269	48%	29	/	60
Celiac lymph nodes	NR				2%	1	/	56	3%	1	/	33	14%	4	/	29
Lymph nodes along the common hepatic artery	<1%	1	/	289	3%	40	/	1483	5%	37	/	774	14%	5	/	37
Splenic artery lymph nodes	NR				2%	1	/	59	0%	0	/	31	26%	11	/	43
Infradiaphragmatic lymph nodes	NR				NR				NR				NR			
Subaortic lymph nodes	NR				10%	2	/	21	0%	0	/	6	NR			
Para-aortic lymph nodes	NR				10%	1	/	10	0%	0	/	3	NR			

NR = not reported. Data presented as percentage of patients with lymph node metastases with number of patients with (number of patients with metastatic lymph nodes/number of patients with lymph node dissection of this station). Only studies that presented data per lymph node station were included in this table.

**Table 5:** Lymph node metastases per pathological T-stage of patients with esophageal carcinoma.

Lymph Node Region	MTE		LTE		UTE		MTE		LTE	
	pT1-2	pT2-3	pT1-2	pT2-3	pT1	pT2-4	pT1	pT2-4	pT1	pT2-4
	*n* = 315	*n* = 915	*n* = 171	*n* = 470	*n* = 22	*n* = 33	*n* = 67	n = 106	*n* = 38	*n* = 90
Cervical region	3%	5%	2%	3%	14%	21%	12%	25%	0%	6%
Upper mediastinal region	3%	6%	2%	5%	55%	85%	22%	61%	13%	27%
Middle mediastinal region	18%	38%	9%	19%	5%	9%	6%	49%	5%	23%
Lower mediastinal region	2%	3%	27%	39%	0%	9%	9%	25%	5%	27%
Abdominal region	11%	16%	25%	33%	NR	NR	NR	NR	NR	NR
Perigastric region	NR	NR	NR	NR	0%	6%	24%	54%	39%	66%
Celiac region	NR	NR	NR	NR	0%	9%	3%	5%	0%	9%

Data presented as percentage of patients with lymph node metastases in this region. UTE = Upper thoracic esophagus; MTE = Middle thoracic esophagus; LTE = Lower thoracic esophagus; NR = not reported.

## Appendix A

**Table A1:** Search strategy for PubMed (22 July 2019).

Search	Query	Items Found
#1	(((((((((((“Esophageal Neoplasms”[Mesh] OR “Esophagectomy”[Mesh] OR ((esophagus[tiab] OR esophageal[tiab] OR esophagogastric[tiab] OR oesophagus[tiab] OR oesophageal[tiab] OR oesophagogastric[tiab] OR gastroesophag*[tiab] OR gastrooesophag*[tiab]) AND (neoplas*[tiab] OR cancer*[tiab] OR carcino*[tiab] OR adenocarcino*[tiab] OR tumor[tiab] OR tumors[tiab] OR tumour[tiab] OR tumours[tiab] OR malig*[tiab])) OR esophagectom*[tiab])) AND (“Lymph Nodes”[Mesh] OR “Lymphatic Metastasis”[Mesh] OR ((lymph[tiab] OR lymphatic[tiab]) AND (node*[tiab] OR nodal[tiab] OR metastas*[tiab])))))) AND ((“Neoplasm Staging”[Mesh] OR staging[tiab] OR TNM[tiab] OR number[tiab] OR extent[tiab] OR extended[tiab] OR scoring[tiab] OR score[tiab] OR classif*[tiab] OR categor*[tiab] OR criteria[tiab] OR 2-field*[tiab] OR two-field*[tiab] OR 3-field*[tiab] OR three-field*[tiab] OR node status[tiab] OR nodal status[tiab] OR D1[tiab] OR D2[tiab] OR N0[tiab] OR N1[tiab] OR N2[tiab] OR N3[tiab] OR pattern*[tiab] OR drainage[tiab] OR spread[tiab] OR pathway*[tiab] OR depth[tiab])))) NOT ((express*[ti] OR overexpress*[ti] OR gene[ti] OR genes[ti] OR protein*[ti] OR p53[ti] OR serum[ti] OR (case[ti] AND report[ti]))) NOT (Animals[Mesh] NOT Humans[Mesh])) AND (english[la] OR dutch[la]))))	4106

[Mesh] = Medical subject headings; [tiab] = words in title OR abstract; [ti] = words in title; [la] = language.

**Table A2:** Search strategy for Embase.com (22 July 2019).

#	Searches	Results
1	exp esophagus tumor/or esophagus resection/or esophagectom*.ti,ab,kw. or ((esophagus or esophageal or esophagogastric or oesophagus or oesophageal or oesophagogastric or gastroesophag* or gastrooesophag*) and (neoplas* or cancer* or carcino* or adenocarcino* or tumor or tumors or tumour or tumours or malig*)).ti,ab,kw.	127708
2	(exp lymph node/or exp lymph node metastasis/or ((lymph or lymphatic) and (node* or nodal or metastas*)).ti,ab,kw.) and (cancer staging/or (staging or TNM or number or extent or extended or scoring or score or classif* or categor* or criteria or 2-field* or two-field* or 3-field* or three-field* or node status or nodal status or D1 or D2 or N0 or N1 or N2 or N3 or pattern* or drainage or spread or pathway* or depth).ti,ab,kw.)	208651
3	1 and 2	10765
4	(express* or overexpress* or gene or genes or protein* or p53 or serum or (case and report)).ti.	2592018
5	animal/ not human/	1423187
6	conference abstract.pt. or conference paper/ or letter/	4893909
7	3 not 4 not 5 not 6	6444
8	limit 7 to (dutch or english)	5195

exp = EMtree keyword with explosion; de = EMtree keyword without explosion; .ab,ti,kw = words in title OR abstract OR keyword; .ti = words in title; .pt = publication type.
